# Loss of SATB2 Occurs More Frequently Than CDX2 Loss in Colorectal Carcinoma and Identifies Particularly Aggressive Cancers in High-Risk Subgroups

**DOI:** 10.3390/cancers13246177

**Published:** 2021-12-07

**Authors:** Maxime Schmitt, Miguel Silva, Björn Konukiewitz, Corinna Lang, Katja Steiger, Kathrin Halfter, Jutta Engel, Paul Jank, Nicole Pfarr, Dirk Wilhelm, Sebastian Foersch, Carsten Denkert, Markus Tschurtschenthaler, Wilko Weichert, Moritz Jesinghaus

**Affiliations:** 1 Institute of Pathology, University Hospital Marburg, 35043 Marburg, Germany; maxime-schmitt@gmx.net (M.S.); paul.jank@uni-marburg.de (P.J.); carsten.denkert@uni-marburg.de (C.D.); 2 Institute of Pathology, Technical University Munich, 81675 Munich, Germany; Bjoern.Konukiewitz@uksh.de (B.K.); corinna.maria.lang@icloud.com (C.L.); katja.steiger@tum.de (K.S.); nicole.pfarr@tum.de (N.P.); wilko.weichert@tum.de (W.W.); 3 II Medizinische Klinik, Klinikum Rechts der Isar, Technical University Munich, 81675 Munich, Germany; gsilva.miguel@gmail.com (M.S.); markus.tschurtschenthaler@tum.de (M.T.); 4 Institute of Pathology, Christian-Albrechts-University, 23562 Kiel, Germany; 5 Munich Cancer Registry (MCR), University Hospital of Munich, Institute for Medical Information Processing, Biometry, and Epidemiology (IBE), Ludwig-Maximilian-University (LMU), 81377 Munich, Germany; halfter@ibe.med.uni-muenchen.de (K.H.); engel@ibe.med.uni-muenchen.de (J.E.); 6 Department of Surgery, Klinikum Rechts der Isar, Technical University Munich, 81675 Munich, Germany; dirk.wilhelm@tum.de; 7 Institute of Pathology, University Hospital Mainz, 55131 Mainz, Germany; sebastian.foersch@unimedizin-mainz.de; 8 Institute for Translational Cancer Research, German Cancer Consortium (DKTK), Partner Site Munich, 81675 Munich, Germany; 9 German Cancer Consortium (DKTK), Partner Site Munich, 81675 Munich, Germany

**Keywords:** SATB2, colorectal carcinoma, prognosis, CDX2

## Abstract

**Simple Summary:**

The immunohistochemical analysis of Special AT-rich sequence-binding protein 2 (SATB2) is increasingly being used to detect colorectal differentiation. Our study aimed to investigate SATB2 expression levels and the prognostic relevance of SATB2 loss in colorectal carcinoma (CRC), especially in comparison with CDX2, the standard marker of colorectal differentiation. We tested SATB2 expression in 1039 CRCs and identified SATB2 as a strong prognosticator in the overall cohort as well as in specific subcohorts, including high-risk subgroups. Compared to CDX2, SATB2 showed a higher prognostic power but was lost at a much higher frequency, generally rendering SATB2 as the less sensitive marker for colorectal differentiation compared to CDX2.

**Abstract:**

Background: Special AT-rich sequence-binding protein 2 (SATB2) has emerged as an alternative immunohistochemical marker to CDX2 for colorectal differentiation. However, the distribution and prognostic relevance of SATB2 expression in colorectal carcinoma (CRC) have to be further elucidated. Methods: SATB2 expression was analysed in 1039 CRCs and correlated with clinicopathological and morphological factors, CDX2 expression as well as survival parameters within the overall cohort and in clinicopathological subgroups. Results: SATB2 loss was a strong prognosticator in univariate analyses of the overall cohort (*p* < 0.001 for all survival comparisons) and in numerous subcohorts including high-risk scenarios (UICC stage III/high tumour budding). SATB2 retained its prognostic relevance in multivariate analyses of these high-risk scenarios (e.g., UICC stage III: DSS: *p* = 0.007, HR: 1.95), but not in the overall cohort (DSS: *p* = 0.1, HR: 1.25). SATB2 loss was more frequent than CDX2 loss (22.2% vs. 10.2%, *p* < 0.001) and of higher prognostic relevance with only moderate overlap between SATB2/CDX2 expression groups. Conclusions: SATB2 loss is able to identify especially aggressive CRCs in high-risk subgroups. While SATB2 is the prognostically superior immunohistochemical parameter compared to CDX2 in univariate analyses, it appears to be the less sensitive marker for colorectal differentiation as it is lost more frequently.

## 1. Introduction

Considering that colorectal carcinoma (CRC) currently ranks among the three most common cancers in humans concerning incidence and mortality worldwide [1,2], further explorations on potentially relevant biomarkers are warranted in order to characterise these tumours precisely and improve prognostic predictions.

Special AT-rich sequence-binding protein 2 (SATB2), a transcription factor interacting with nuclear matrix attachment regions which is highly expressed in the non-neoplastic colorectal mucosa [3,4], attracted increasing scientific notice for the identification of the colorectal origin of cancers of unknown primary and of CRC metastases [5,6,7,8,9,10], delineating SATB2 as a valid addition to CDX2, which is still the most established immunohistochemical marker associated with colorectal differentiation [5,11].

Previous immunohistochemical assessments of SATB2 in CRC showed a general association of a diminished SATB2 expression with poorer survival characteristics and microsatellite status [12,13,14,15,16]. However, it remains unclear whether SATB2 is differently expressed within purely morphological (adenocarcinoma NOS vs. specific CRC subtypes, tumour budding subcategories (Bd1/2/3), WHO low- vs. high-grade carcinomas), immunohistochemical (CDX2 expression) and pTNM/UICC stage subgroups. Furthermore, it remains to be elucidated, how frequently the loss of SATB2 occurs in comparison to the loss of CDX2 and whether SATB2 can identify distinct prognostic groups within these colorectal cancer subsets.

To address these questions, we investigated SATB2 expression in a large cohort comprising 1039 CRCs and correlated the results with histomorphologic and immunohistochemical (CRC subtypes, tumour budding activity, WHO grade, CDX2 expression) as well as clinicopathological parameters (pTNM/UICC staging, tumour localisation) and explored the prognostic relevance of SATB2 expression in uni- and multivariate survival analyses in the overall cohort as well as in specific subgroups.

## 2. Materials and Methods

### 2.1. Study Population

Our study cohort comprised one thousand and thirty-nine CRC patients that underwent surgical resection between 1997 and 2019 at the University Hospital Klinikum rechts der Isar of the Technical University of Munich, Germany. Only patients with colorectal carcinomas were included in this study. Patients suffering from other colorectal tumours (e.g., neuroendocrine tumours, non-epithelial tumours, etc.), insufficient tissue on the constructed tissue microarray or incomplete clinicopathological/survival data were excluded. The original cohort (1997–2018) was recently extended with cases from 2019 (*n* = 36) [17]. Clinicopathological characteristics as well as survival data were extracted from hospital records or from the Munich Cancer Registry. Definitions of survival parameters, survival endpoints and general treatment modalities were defined as described previously [18,19]. The local ethic committee of the Technical University of Munich approved this study (reference number: 252/16 s).

### 2.2. Evaluation of SATB2 Expression and Clinicopathological Parameters

SATB2 expression was analysed by SATB2 immunohistochemistry in 1039 CRCs on a tissue microarray with two tumour-carrying cores from each tumour. We used an automated immunostainer (BOND RXm System, Leica Biosystems, Germany) for the immunohistochemical staining of SATB2 (EP281, Cellmarque 384R, Ready-To-Use, Cell Marque, USA), which is the standard SATB2 antibody used in daily clinicopathological routine and which has been used by various previous studies [5,11,15,20]. We stained 2 µm thick sections from our tissue microarray. Antigen retrieval was performed with Epitope Retrieval 1 after deparaffinisation, (Leica Biosystems, Germany; equivalent to citrate buffer pH 6) for 20 min and antibody binding was detected using a Polymer Refine Detection Kit (Leica Biosystems, Nußloch, Germany) without a postprimary antibody and haematoxylin counterstain. Naturally, pretested positive/negative control-tissues were stained in parallel. Two independent observers (MJ, MS (Maxime Schmitt)) that were blinded to clinicopathological parameters manually performed the evaluation of SATB2 expression.

We assessed the number of positive carcinoma cells for each individual patient. Counting a minimum of 500 tumour cells, the resulting cumulative percentage score for both cores was then assigned for each CRC (range: 0–100%). Only a nuclear staining of SATB2 was considered specific. SATB2 expression patterns (combined from both cores) were classified into three separate groups: diffuse, if the tumours either showed a complete expression or only a very focal loss in singular cells; heterogeneous, if areas with a complete loss of staining were observed; absent, if the tumours were completely negative. A strong staining intensity was defined as an intensity comparable to normal colonic mucosa, a still easily identifiable but slightly weaker staining was rated as medium. A barely visible staining intensity was classified as weak. Cases without any detectable staining were classified as absent.

SATB2-grouping results from the TMA were compared with SATB2 staining from 20 randomly selected full block slides, interobserver variability was probed in 150 cases that were assessed by the two observers in a blinded fashion.

SATB2 expression was correlated with clinicopathological variables including staging data and with Haematoxylin and Eosin-based histopathological parameters defined by the recent WHO classification (CRC subtypes: Adenocarcinoma NOS, Mucinous adenocarcinoma, Signet-ring carcinoma, Medullary carcinoma, Serrated adenocarcinoma, Micropapillary adenocarcinoma, Adenoma-like adenocarcinoma, Adenosquamous carcinoma, Carcinoma with sarcomatoid components, Undifferentiated carcinoma, MANEC/NEC; WHO grade: low grade, formerly G1/G2 vs. high grade, formerly G3 and tumour budding: Bd1 (0-4 Buds in 20×), Bd2 (5-9 Buds in 20×), Bd3 (≥10 Buds in 20×)). The parameters were available from a previous study on the same cohort regarding the distribution and the prevalence of the essential morphologic criteria given in the 2019 WHO classification of colorectal carcinoma, from which also the microsatellite status was extracted [17] (cohort details; Table 1). Furthermore, SATB2 expression was correlated with CDX2 expression, which was analysed in a previous study [18], where a similar methodology regarding the finding of an optimised cutoff for CDX2 expression groups was used [21]. The cases from 2019 that were recently added to the collective (*n* = 36) were classified regarding the aforementioned parameters (histomorphology, CDX2 expression) as described previously [17,18].

### 2.3. Statistics

Using SPSS version 26 (SPSS Institute, Chicago, IL, USA) statistical analyses were performed applying X^2^ test as well as X^2^ test for trends and Fisher’s exact test. The Cutoff Finder, a publicly available biostatistical tool that represents a bundle of optimisation and visualisation methods for cutoff determination, was used to define the optimal cutoffs for SATB2 expression groups [21]. Where applicable, the Bonferroni method was used to correct for multiple testing. Univariate survival analyses were performed using the Kaplan–Meier method and significance of survival differences was tested by a log-rank test. The Cox proportional hazard model was used for multivariate analyses. All statistical tests were performed two-sided, *p*-values ≤ 0.05 were considered significant.

## 3. Results

### 3.1. Clinicopathological Features and Survival

The median patient age was 69 years. The majority of patients were male (*n* = 599; 58%). Left- (descending colon until rectum; *n* = 536; 52%) and right-sided (coecum until splenic flexure; *n* = 503; 48%) neoplasms showed an almost even distribution. Postoperative UICC staging (eighth edition of the TNM classification of malignant tumours) [22] resulted in 213 stage I (21%), 350 (34%) stage II, 318 (31%) stage III and 158 (15%) stage IV cancers. Three hundred and thirty patients (32%) relapsed, 411 patients (40%) died during follow up, for 301 (29%) patients a tumour-specific death was noted (cohort details: Table 1).

#### 3.1.1. Distribution of SATB2 Expression and Biostatistical Generation of SATB2 Expression Groups

**Figure 1:**
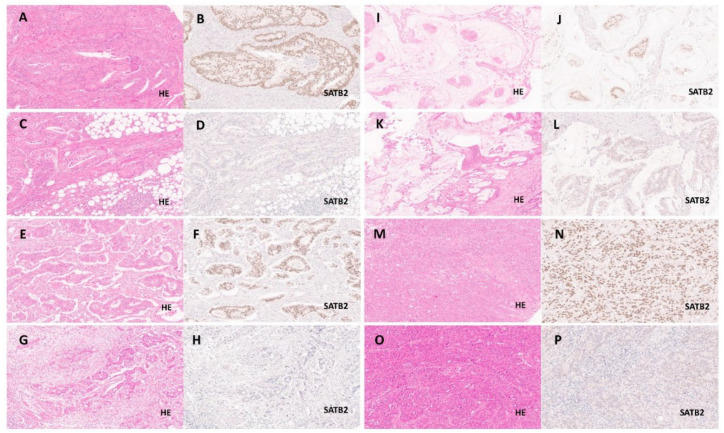
SATB2-low/absent and SATB2-high expression groups in selected colorectal carcinoma subtypes. (**A**–**D**): Adenocarcinoma NOS from the SATB2-high ((**A**): HE, 20× + (**B**): SATB2, 20×) and from association the SATB2-low/absent ((**C**): HE, 20× + (**D**): SATB2, 20×) expression subgroup. (**E**–**H**)**:** Micropapillary adenocarcinoma from the SATB2-high ((**E**): HE, 20× + (**F**): SATB2, 20×) and from association the SATB2-low/absent ((**G**: HE, 20× + (**H**: SATB2, 20×) expression subgroup, also shown as an example of carcinomas with a high tumour budding activity from both expression groups. (**I**–**L**): Mucinous adenocarcinoma from the SATB2-high ((**I**): HE, 20× + (**J**): SATB2, 20×) and from association the SATB2-low/absent ((**K**): HE, 20× + (**L**): SATB2, 20×) expression subgroup. (**M**–**P**): Medullary carcinoma from the SATB2-high ((**M**): HE, 20× + (**N**): SATB2, 20×) and from association the SATB2-low/absent ((**O**): HE, 20× + (**P**): SATB2, 20×) expression subgroup.

Most CRCs showed a diffuse SATB2 expression (61%, *n* = 639), a heterogeneous staining was noted for 340 cancers (33%), 60 tumours (6%) showed a complete absence of SATB2. A nuclear staining in ≥90% of tumour cells was observed in 65% (679/1039) of cases. In order to transform this continuous variable into dichotomous SATB2 groups (binary variable), we used the Cutoff Finder [21], a publicly available biostatistical tool for cutoff determination, to identify the best cutoff for SATB2 stratification. Following these initial statistical analyses, two SATB2-groups were formed: CRCs that showed an SATB2 expression above the 20th percentile (>70% tumour cells; *n* = 808, 78%) were categorised as SATB2-high, CRC on/below the 20th percentile (range:0–70% of tumour cells; *n* = 231, 22%) were categorised as SATB2-low/absent. Examples of the two SATB2 expression groups among certain CRC subtypes are given in Figure 1. SATB2-low/absent CRCs usually showed a reduced SATB2 staining intensity and a significantly higher rate of a heterogeneous/absent staining pattern (*p* < 0.001, details see Table S1). Only the number of positive tumour cells (regardless of staining pattern or intensity) were used to form the SATB2 expression groups. A comparison of the results of the SATB2-grouping with full block slides showed an excellent concordance with the results from the TMA (95%, *p* < 0.001, Kappa Cohens value: 0.88). Furthermore, an excellent interobserver variance was evident (*p* < 0.001, Kappa Cohens value: 0.95).

#### 3.1.2. Association of SATB2-Groups with pTNM/UICC Staging, Morphologic Parameters (CRC Subtypes/Tumour Budding/WHO Grade) and Microsatellite Status

**Figure 2:**
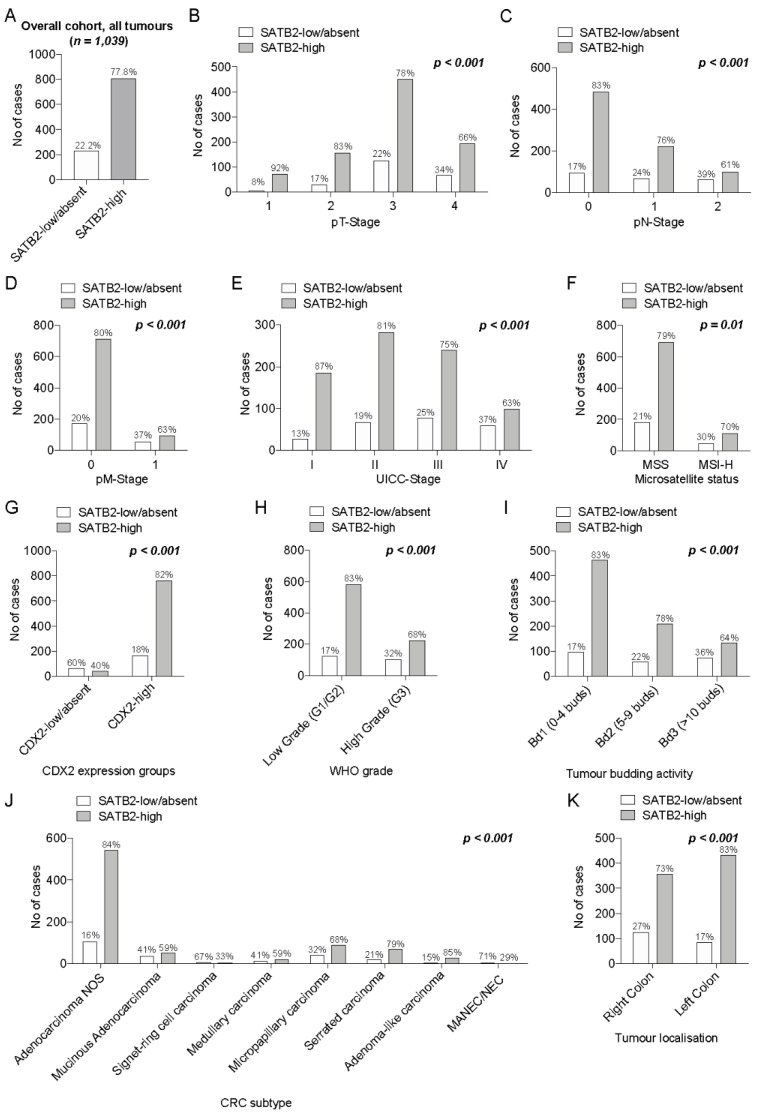
(**A**–**D**): Prevalence of SATB2 expression groups within the overall cohort (**A**) and in pT (**B**), pN (**C**), pM (**D**) and combined UICC stage subgroups (**E**). (**F**,**G**): Distribution of SATB2 expression groups among MSS vs. MSI-H (**F**) and CDX2-low/absent vs. CDX2-high CRCs (**G**). (**H**–**K**): Differential distribution of SATB2 within WHO low-grade vs. WHO high-grade CRCs (**H**), among the different tumour budding subgroups (Bd1, Bd2, Bd3; (**I**)), among colorectal cancer subtypes (**J**) and in right- vs. left-sided CRCs (**K**).

As illustrated in Figure 2 and depicted in detail in Table S2, SATB2-low/absent CRCs were significantly enriched in higher pT/pN/pM and combined UICC-stages, right-sided tumours, carcinomas with lymphatic and blood vessel invasion as well as in tumours with positive margins (*p* < 0.001, respectively). Compared to SATB2-high neoplasms, SATB2-low/absent CRCs were significantly increased in CRCs with high (Bd3) tumour budding activity and in poorly differentiated carcinomas according to the WHO grade (*p* < 0.001, respectively). Furthermore, a low/absent SATB2 expression was significantly enriched in the mucinous, micropapillary, medullary and signet-ring CRC subtypes as well as in MANEC/NEC (*p* < 0.001). MSI-H CRCs were also associated with an absent or low SATB2 expression (*p* = 0.01).

#### 3.1.3. Association of SATB2 Expression Groups with CDX2 Expression Groups

Far more CRCs were allocated to the SATB2 low/absent expression group than to the CDX2 low/absent expression group (CDX2: 10.2% vs. SATB2: 22.2%). Although both expression groups were associated with one another (*p* < 0.001), there were many tumours with a discordant SATB2/CDX2 expression status (Kappa Cohens value: 0.30). For example, only 28% (64/231) of CRCs from the SATB2-low/absent subgroup showed a concordant low/absent CDX2 expression, while 40% (42/106) of CDX2-low/absent CRCs showed a high SATB2 expression level. Sixty CRCs (6%) showed a complete absence of SATB2 expression compared to only 13 CRCs (1.3%) that showed a complete negativity for CDX2. Only two CRCs remained negative for both markers, while the rest of the CRCs without any SATB2 expression showed a heterogeneous (*n* = 19, 32%) or diffuse expression of CDX2 (*n*= 39, 65%). Of the 13 completely CDX2 negative cases, the vast majority (*n* = 11, 85%) showed a heterogenous or diffuse SATB2 expression (details: Table 2).

### 3.2. Prognostic Relevance of SATB2-Groups in the Overall Cohort

**Figure 3:**
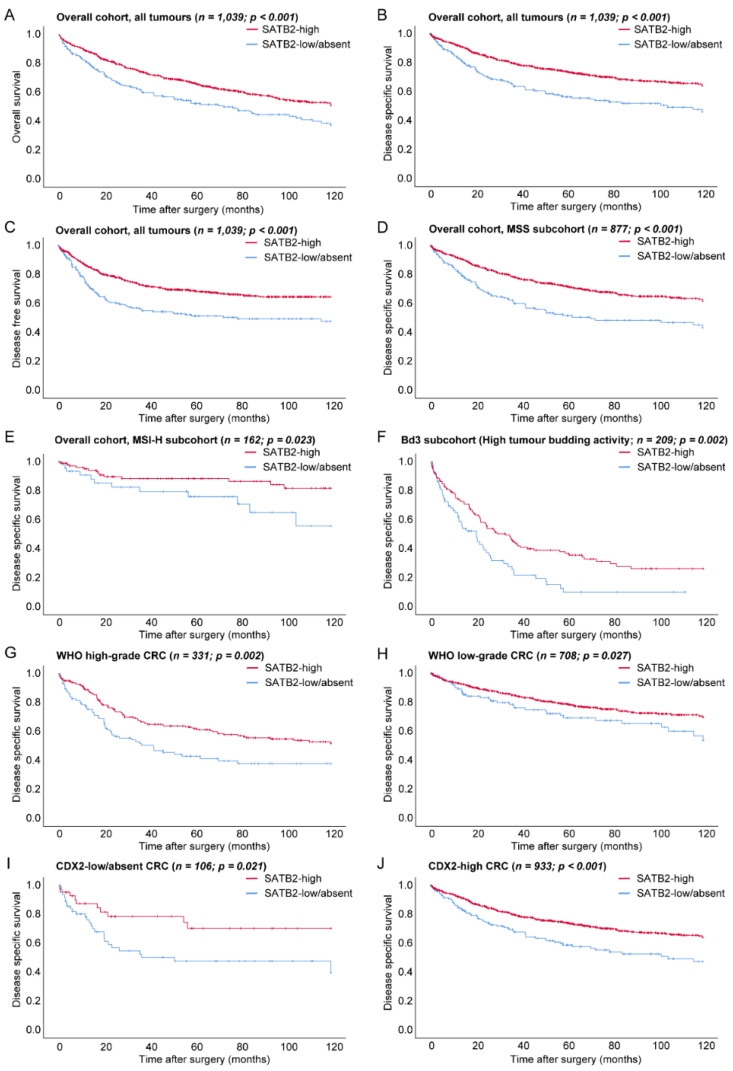
Prognostic relevance of SATB2 expression in univariate analyses on overall-, disease specific- and disease free-survival in the overall cohort (**A**–**C**) and for disease-specific survival in specific CRC subgroups: MSS (**D**) and MSI-H (**E**) subcohorts, high tumour budding activity subcohort (**F**), WHO high-grade (**G**)/WHO low-grade (**H**) subcohorts, CDX2-low/absent (**I**)/CDX2-high (**J**) subcohorts.

As illustrated in Figure 3 and Table 1, compared to SATB2-high CRCs, the SATB2-low/absent group showed a significantly decreased OS (SATB2-high 82.2 months vs. SATB2-low/absent 68.4 months, *p* < 0.001), DSS (SATB2-high 91.5 months vs. SATB2-low/absent 74.2 months, *p* < 0.001) and DFS (SATB2-high 86.3 months vs. SATB2-low/absent 68 months, *p* < 0.001) in univariate analyses (log-rank test) of the overall cohort of 1039 CRCs.

#### 3.2.1. Prognostic Relevance of SATB2 in Microsatellite and CDX2 Expression Subgroups

A strong prognostic impact of the SATB2-low/absent group on OS/DSS/DFS was present in MSS CRCs (65.4 months/70 months/62.8 months, *p* < 0.001, respectively). SATB2 was also prognostic for DSS (SATB2-high 105.8 months vs. SATB2-low/absent 90.6 months, *p* = 0.023), but not for OS (SATB2-high 92.3 months vs. SATB2-low/absent 78.8 months, *p* = 0.072) or DFS (SATB2-high 101 months vs. SATB2-low/absent 89 months, *p* = 0.163) in MSI-H CRC.

When analysed in CDX2 expression subgroups, SATB2 expression groups showed a strong prognostic demarcation in both CDX2-low/absent (e.g., DSS: SATB2-high 91.2 months vs. SATB2-low/absent 64.5 months, *p* = 0.021) as well as in CDX2 high CRCs (e.g., DSS: SATB2-high 91.6 months vs. SATB2-low/absent 77.3 months, *p* < 0.001) (Figure 3, Table S3), while CDX2 expression showed no prognostic relevance in any of the SATB2 expression groups (e.g., *p* > 0.05, data not shown).

#### 3.2.2. Prognostic Relevance of SATB2 in WHO Grade and Tumour Budding Subgroups

SATB2 expression showed a strong prognostic impact in WHO low-grade (e.g., DSS: SATB2-high 96.7 months vs. SATB2-low/absent 88.1 months, *p* = 0.027) and high-grade CRCs (e.g., DSS: SATB2-high 78.8 months vs. SATB2-low/absent 59.5 months, *p* = 0.002).

When analysed within the different tumour budding subgroups (Bd1/2/3), SATB2 showed a weak prognostic relevance in tumours with a low tumour budding activity (e.g., DSS: SATB2-high 110.2 months vs. SATB2-low/absent 104.4 months *p* = 0.05) and especially a strong prognostic impact within CRCs with a high tumour budding activity, where CRCs with a low/absent SATB2 expression showed a significantly worse survival rate compared to SATB2-high carcinomas (e.g., DSS: SATB2-high 51.1 months vs. SATB2-low/absent 29 months, *p* = 0.002) (Figure 3, Table S3).

#### 3.2.3. Prognostic Relevance of SATB2 in UICC Stage Subgroups and Right vs. Left-Sided CRCs

In UICC stage III tumours, SATB2-low/absent showed significantly shortened survival characteristics (OS: 70.3 months vs. 84.4 months, *p* = 0.025; DSS: 74.5 months vs. 91 months, *p* = 0.012; DFS: 62.1 months vs. 81.2 months; *p* = 0.004) (Table S4) in all survival comparisons. In UICC stage I/II and IV, no significant survival impact was visible.

SATB2 expression showed a strong prognostic impact in right-sided (e.g., DSS: SATB2-high 90.8 months vs. SATB2-low/absent 77.4 months, *p* = 0.002) and left-sided CRCs (e.g., DSS: SATB2-high 92.1 months vs. SATB2-low/absent 68.9 months, *p* < 0.001) (Figure S1).

### 3.3. Multivariate Analyses

In multivariate analyses (including age, gender, resection status, UICC stage, MSI-status, WHO grade, tumour budding, CRC subtypes and SATB2-groups) SATB2-expression was not an independent prognostic factor (e.g., DSS: *p* = 0.1, hazard ratio: 1.25, Table S5) in the overall cohort comprising all CRCs. When the other main histological confounders (WHO grade, tumour budding, CRC subtypes) were excluded from the Cox-regression analysis, SATB2-expression remained a prognostic factor independent of UICC stage, age, gender, resection status and MSI-status (DSS: 0.029, HR:1.32, Table S6).

In a full multivariate analyses (including all parameters mentioned above) of high risk CRC subcohorts (UICC stage III/high tumour budding activity), SATB2 fully retained its prognostic relevance demonstrated in univariate analyses (UICC stage III CRC subcohort: DSS: *p* = 0.007, hazard ratio: 1.95, Table 3; Bd3-CRCs with a high tumour budding activity DSS: *p* = 0.01, hazard ratio: 1.67, Table 4; DFS: *p* = 0.02, Hazard Ratio: 1.79; OS: *p* = 0.01, hazard ratio: 1.58, data not shown).

## 4. Discussion

In this study, we investigated the expression of Special AT-rich sequence-binding protein 2 (SATB2) in one thousand and thirty-nine resected CRCs, correlated the results with histomorphologic parameters (CRC subtypes, tumour budding activity, WHO grade) [17], expression of CDX2 [18] as well as clinicopathological parameters (pTNM/UICC staging, microsatellite status, localisation) and analysed the prognostic relevance of SATB2 in the overall cohort and in specific subcohorts.

In recent years, SATB2 has gained increasing attention as a relatively specific marker of colorectal differentiation [23,24,25,26] and functional studies have revealed the tumour-suppressive properties of SATB2 in experimental settings [12,27,28,29,30], demonstrating that SATB2 is a complexly regulated tumour suppressor that represses CRC progression by inhibiting the transcription of SNAIL, a master regulator of epithelial-mesenchymal transition. In our cohort of more than one thousand tumours, SATB2 low/absent CRCs were significantly associated with higher UICC stages and massively enriched in tumours with high-risk histomorphological features such as high tumour budding (Bd3) or poorly differentiated carcinomas according to the WHO grade, which is in line with the functional studies postulating the tumour suppressive properties of SATB2 [12,27,28]. Consistent with previous findings, SATB2-low/absent CRCs were also associated with strongly reduced survival parameters in univariate analyses (log-rank test) of the overall cohort. Interestingly, although SATB2 expression retained its statistical significance in multivariate analyses when UICC stage, resection status, MSI-status, age and gender were incorporated, this independent prognostic power vanished when we added the most common histomorphological parameters of CRC, tumour budding, WHO grade and the different CRC subtypes to our multivariate analyses. These findings argue towards the fact that the massive enrichment of SATB2 low expressing tumours in high-grade categories of these parameters probably washes out the strong prognostic effect of SATB2 that is present in univariate analyses.

Nevertheless, we wanted to know whether SATB2 can identify any prognostic subgroup that is not identified by either UICC staging or classical histomorphological parameters and which is also retained in multivariate analyses incorporating all factors. Interestingly, SATB2 low/absent CRCs were associated with an especially aggressive disease course in CRCs with high tumour budding activity and in UICC stage III cancers and showed highly reduced survival times in these high-risk subgroups. In subsequent multivariate analyses incorporating all prognostic factors, SATB2 retained its prognostic relevance in both UICC stage III carcinomas and dissociative cancers with a high tumour budding activity. These findings delineate SATB2 as a worthwhile immunohistochemical biomarker in CRC that can identify especially aggressive cancers in these high-risk subgroups of CRC and delivers valuable additional prognostic information in addition to standard histomorphological factors and UICC staging. Regarding the translation of these results into clinicopathological routine, we suggest that the responsible pathologist reports SATB2 loss by stating the percentage of SATB2 expressing cancer cells, ideally combined with the notion that a significantly reduced expression of SATB2 has been associated with a poorer clinical outcome.

As SATB2 has emerged as a considerable alternative to CDX2 to verify or rule out colorectal differentiation, another aim of our study was to compare the incidence and overlap of SATB2 loss with loss of CDX2 and also to compare the prognostic relevance of these two markers with one another [18]. The findings of these aspects of our study are also particularly interesting, because they highlight possible strengths and weaknesses of the most commonly used colorectal markers. Notably, the frequency of a reduced or completely lost SATB2 expression is much higher compared to CDX2 and especially a completely absent SATB2 expression was far more frequent than a complete negativity for CDX2. This implies, that when both CDX2 and SATB2 (as singular markers) are assessed regarding their ability to detect a colorectal origin in neoplastic tissues, SATB2 has to be ranked as the less sensitive marker compared to CDX2. However, as only two out of 1039 CRCs (0.2%) showed a complete loss of both SATB2 and CDX2, and the majority of SATB2 negative CRCs showed a strong and diffuse expression of CDX2 (and vice versa), a combined panel of both markers appears to be able to identify the overwhelming majority of colorectal cancers and is probably the most expedient approach for routine diagnostic settings.

When we then moved on to compare the prognostic impact of SATB2 and CDX2, we observed an opposing picture. Compared to CDX2, the loss of SATB2 showed a considerably higher prognostic impact in univariate analyses (log-rank test) of the overall cohort and in nearly all clinicopathological subscenarios of CRC, in which CDX2 mostly showed at best minimal prognostic impact in our cohort. A crucial difference between the prognostic difference of SATB2 and CDX2 was also that SATB2 retained its prognostic power in right- and left-sided CRCs, while CDX2 did not show any prognostic significance in right-sided tumours although it is more frequently lost in the right colon [18]. In line with these findings, we additionally observed that SATB2-low/absent CRCs were able to identify patients with a poor prognosis in both CDX2 expression groups (CDX2-low/absent vs. CDX2- high), while CDX2 showed no prognostic relevance in SATB2 expression subgroups, rendering SATB2 as the prognostically superior immunohistochemical biomarker in CRC compared to CDX2.

## 5. Conclusions

Our study has five major findings: (1) a low/absent SATB2 expression is significantly enriched in advanced stage CRCs that have an aggressive histomorphological phenotype with high tumour budding activity and/or a poor differentiation according to the WHO grade. (2) Loss of SATB2 is of high prognostic relevance in uni- and multivariate analyses (including UICC stage) in the overall cohort, but shows no independent prognostic value in the overall cohort when the main histomorphological parameters of CRC (tumour budding, WHO grade, CRC subtypes) are added to the multivariate analyses. (3) SATB2 shows an especially high prognostic relevance in uni- and multivariate analyses of high-risk clinicopathological subgroups (high tumour budding/UICC stage III) and identifies CRCs with a particularly aggressive disease course in these high-risk scenarios. (4) SATB2 loss occurs much more frequently than loss of CDX2, with a substantial portion of SATB2-negative CRCs showing a diffuse or at least heterogeneous CDX2 positivity, generally delineating CDX2 as the more sensitive marker of colorectal differentiation in carcinomas. (5) SATB2, in general, showed a vastly better prediction of survival outcome compared to CDX2, with SATB2 retaining its prognostic impact in CDX2 expression subgroups (CDX2 low/absent vs. high), rendering SATB2 as the superior prognostic biomarker compared to CDX2.

In conclusion, our study identifies SATB2 as a potentially valuable additional prognostic biomarker in CRC. Further studies are warranted to explore the possible therapeutic implications of a diminished or completely lost SATB2 expression. Both SATB2 and CDX2 can individually be completely lost in CRCs, while a total absence of both markers is almost never observed. Therefore, a combined panel of both markers appears to be the most solid approach to pinpoint or rule out colorectal differentiation.

## Figures and Tables

**Table 1:** Distribution and prognostic impact of SATB2-expression and clinicopathological parameters in the overall cohort.

Variables		Overall *n* (%)	Median Overall Survival (SE) (Months)	*p*-Value	Mean Disease Specific Survival (SE) (Months)	*p*-Value	Mean Disease Free Survival (SE) (Months)	*p*-Value
Age				<0.001		0.02		0.98
	below median	504 (48.5%)	86.4 (2.2)		91.2 (2.1)		82.4 (2.3)	
	above median	535 (51.5%)	72.1 (2.2)		84.2 (2.3)		82.7 (2.4)	
Sex				0.33		0.93		0.54
	male	599 (57.7%)	78.1 (2.1)		88 (2.0)		83.6 (2.2)	
	female	440 (42.3%)	80.7 (2.4)		87.4 (2.4)		81 (2.6)	
SATB2 Subgroups				<0.001		<0.001		<0.001
	SATB2-low/absent	231 (22.2%)	68.4 (3.6)		74.2 (3.6)		68 (3.9)	
	SATB2-high	808 (77.8%)	82.2 (1.7)		91.5 (1.7)		86.3 (1.8)	
pT				<0.001		<0.001		<0.001
	1	79 (7.6%)	97.7 (4.8)		115.5 (3.0)		109.8 (3.6)	
	2	187 (18%)	92.8 (3.2)		103.9 (2.8)		100 (3.1)	
	3	578 (55.6%)	79.7 (2.1)		88.3 (2.1)		82.2 (2.2)	
	4	195 (18.8%)	57.2 (3.7)		60.4 (3.8)		55 (4.0)	
pN				<0.001		<0.001		<0.001
	0	580 (55.8%)	89 (1.9)		101.2 (1.7)		99.1 (1.8)	
	1	292 (28.1%)	75.7 (3.0)		81 (3.0)		73.2 (3.2)	
	2	167 (16.1%)	51.4 (4.0)		54.6 (4.2)		42.6 (4.0)	
pM				<0.001		<0.001		<0.001
	0	887 (85.4%)	86.5 (1.6)		96.3 (1.5)		91.1 (1.7)	
	1	152 (14.6%)	40.1 (3.5)		42.7 (3.7)		34.4 (3.6)	
UICC Stage				<0.001		<0.001		<0.001
	1	213 (20.5%)	96.6 (2.9)		111.1 (2.1)		107.8 (2.4)	
	2	350 (33.7%)	86 (2.6)		97 (2.4)		95.7 (2.6)	
	3	318 (30.6%)	81.2 (2.8)		87.2 (2.8)		76.6 (3.1)	
	4	158 (15.2%)	39.3 (3.4)		41.8 (3.6)		33.3 (3.5)	
Tumour type (WHO)				<0.001		<0.001		<0.001
	Adenocarcinoma NOS	650 (62.6%)	83.7 (1.9)		92.5 (1.9)		87.4 (2.0)	
	Mucinous adenocarcinoma	88 (8.5%)	76.5 (5.5)		87 (5.6)		78.1 (6.0)	
	Signet-ring cell carcinoma	9 (0.8%)	54 (22.5)		54 (22.5)		34.4 (18.9)	
	Medullary adenocarcinoma	32 (3.1%)	98.6 (7.2)		116.3 (3.6)		112.8 (4.9)	
	Micropapillary adenocarcinoma	129 (12.4%)	53.6 (4.4)		56.2 (4.6)		47.3 (4.5)	
	Serrated adenocarcinoma	91 (8.7%)	78.4 (5.6)		87.7 (5.4)		84.4 (5.6)	
	Adenoma-like adenocarcinoma	33 (3.2%)	98 (6.4)		115.2 (3.5)		116.4 (3.5)	
	MANEC/NEC	7 (0.7%)	18 (8.2)		18.0 (8.1)		15.8 (8.4)	
WHO grade				<0.001		<0.001		<0.001
	low-grade	708 (68.1%)	86 (1.8)		95.2 (1.7)		89.6 (1.9)	
	high-grade	331 (31.9%)	65.4 (2.8)		72.6 (2.9)		67.8 (3.1)	
Tumour				<0.001		<0.001		<0.001
budding	Bd1	560 (53.9%)	97.8 (1.7)		109.3 (1.3)		107 (1.5)	
	Bd2	270 (26%)	70.5 (3.1)		77.6 (3.1)		66.8 (3.3)	
	Bd3	209 (20.1%)	41 (3.1)		44 (3.4)		36.6 (3.4)	
Resection				<0.001		<0.001		<0.001
margin	R0	960 (92.4%)	83.0 (1.6)		92.4 (1.5)		87.4 (1.7)	
	R1	49 (4.7%)	40.8 (7.2)		42.3 (7.4)		29.2 (6.1)	
	R2	30 (2.9%)	25.0 (4.5)		25 (4.5)		21.5 (3.7)	
Lymphatic				<0.001		<0.001		<0.001
invasion	not present	508 (48.9%)	89.7 (2.0)		101.8 (1.8)		100.6 (1.9)	
	present	531 (51.1%)	69 (1.6)		74.2 (2.3)		65.2 (2.5)	
Venous				<0.001		<0.001		<0.001
invasion	not present	904 (87%)	83.7 (1.6)		93.4 (1.6)		89.2 (1.7)	
	present	135 (13%)	48.7 (4.3)		50.9 (4.4)		38.6 (4.1)	
Microsatellite				0.01		0.001		<0.001
status	Microsatellite stable	877 (84.4%)	77.6 (1.7)		85.5 (1.7)		80 (1.8)	
	Microsatellite instable	162 (15.6%)	88.7 (3.8)		101.4 (3.3)		97.8 (3.7)	
CDX2				0.012		0.006		0.012
subgroups	CDX2-low/absent	106 (10.2%)	67.9 (5.6)		75.8 (5.7)		70.4 (5.9)	
	CDX2-high	933 (89.8%)	80.4 (1.6)		89.1 (1.6)		83.7 (1.7)	
Tumour				0.26		0.83		0.93
localization	Right (Coec/Asc/Trans)	503 (48.4%)	77.2 (2.3)		87.1 (2.3)		82.7 (2.4)	
	Left (Desc/Sigm/Rect)	536 (51.6%)	81.1 (2.1)		88.4 (2.1)		82.3 (2.3)	

**Table 2:** Correlation of SATB2 and CDX2 expression groups and CDX2/SATB2 staining patterns.

Variables						Total	*p*-Value
A	SATB2 expression group						
CDX2 expression group		low/absent		high			
	low/absent	64		42		106	*p* < 0.001
	high	167		766		933	
total		231		808		1039	
B	CDX2 staining pattern						
SATB2 staining pattern		Absent	heterogenous		diffuse		
	absent	2	19		39	60	*p* < 0.001
	heterogenous	7	70		263	340	
	diffuse	4	32		603	639	
total		13	121		905	1039	

**Table 3:** Multivariate disease-specific survival analysis in the UICC stage III subcohort under inclusion of SATB2 expression, age, gender, CRC subtype, tumour budding, WHO grade, resection status and microsatellite status.

Variables		HR (DSS)	Lower CI (95%)	Upper CI (95%)	*p*-Value
SATB2 subgroups					0.007
	SATB2 high	1.00			
	SATB2 Low/absent	1.95	1.20	3.16	
WHO Subtype					0.026
	Adenocarcinoma NOS	1.00			
	Mucinous adenocarcinoma	0.41	0.12	1.34	
	Signet-ring cell carcinoma	1.89	0.53	6.70	
	Medullary carcinoma	0.17	0.02	1.40	
	Micropapillary adenocarcinoma	0.75	0.43	1.29	
	Serrated adenocarcinoma	0.82	0.40	1.68	
	Adenoma-like adenocarcinoma	0.00	0.00	>30	
	MANEC/NEC	5.67	1.23	26.04	
Tumour budding					<0.001
	Bd1	1.00			
	Bd2	3.35	1.76	6.36	
	Bd3	5.78	2.95	11.34	
WHO-grade					0.027
	Low grade	1.00			
	High grade	1.63	1.06	2.51	
Gender					0.723
	female	1.00			
	male	1.08	0.69	1.69	
Resection status					0.001
	R0	1.00			
	R1/2	2.72	1.47	5.04	
Tumour					0.812
localization	Right colon	1.00			
	Left colon	0.94	0.59	1.52	
Age group					0.112
	Below median	1.00			
	Median and above	1.43	0.92	2.34	
Microsatellite					0.817
status	Microsatellite instable	1.00			
	Microsatellite stable	1.09	0.51	2.32	

**Table 4:** Multivariate disease-specific survival analysis in the high tumour budding (Bd3) subcohort under inclusion of SATB2 expression, age, gender, CRC subtype, UICC stage, WHO grade, resection status and microsatellite status.

Variables		HR (DSS)	Lower CI (95%)	Upper CI (95%)	*p*-Value
SATB2 subgroups					0.01
	SATB2 high	1.00			
	SATB2 Low/absent	1.67	1.13	2.46	
WHO Subtype					0.1
	Adenocarcinoma NOS	1.00			
	Mucinous adenocarcinoma	1.49	0.70	3.16	
	Signet-ring cell carcinoma	1.30	0.44	3.80	
	Micropapillary adenocarcinoma	0.80	0.54	1.19	
	Serrated adenocarcinoma	0.90	0.43	1.90	
	MANEC/NEC	2.31	0.85	6.25	
WHO-grade					0.067
	Low grade	1.00			
	High grade	1.43	0.97	2.09	
UICC Stage					0.006
	I	1.00			
	II	0.67	0.24	1.85	
	III	0.64	0.24	1.70	
	IV	1.41	0.53	3.75	
Gender					0.716
	female	1.00			
	male	1.07	0.74	1.55	
Resection status					0.013
	R0	1.00			
	R1/2	1.44	1.08	1.91	
Tumour					0.108
localization	Right colon	1.00			
	Left colon	1.34	0.94	1.93	
Age group					0.767
	Below median	1.00			
	Median and above	1.06	0.72	1.57	
Microsatellite					0.159
status	Microsatellite instable	1.00			
	Microsatellite stable	1.76	0.80	3.88	

## Data Availability

All relevant data are within the paper and its supporting information files. The data underlying the results presented in the study are available from the study group upon reasonable request, some restrictions apply due to confidentiality of patient data. Since these data are derived from a research trial with ongoing follow up there are legal and ethical restrictions to share sensitive patient related data publicly. Data can be requested in the context of a translational research project by sending a request to the corresponding author.

## Supplementary Materials

The following are available online at https://www.mdpi.com/article/10.3390/cancers13246177/s1. Figure S1: Prognostic relevance of SATB2 expression for disease-specific survival in right-sided (A, coecum until splenic flexure) and left-sided (descending colon until rectum) CRCs, Table S1: Correlation between SATB2 expression groups with SATB2 staining pattern (A) and SATB2 staining intensity (B), Table S2: Distribution of SATB2 expression groups with clinicopathological and morphological parameters in the overall cohort, Table S3: Impact of SATB2 expression on overall, disease-specific and disease-free survival in tumour budding—(Bd1, Bd2, Bd3), WHO grade (low, high), microsatellite (MSS, MSI-H) and CDX2 expression subcohorts (low/absent, high), Table S4: Impact of SATB2 expression on overall, disease-specific and disease-free survival in the different UICC stage groups, Table S5: Multivariate overall survival analysis in the overall cohort under inclusion of SATB2 expression, age, gender, CRC subtype, tumour budding, WHO grade and microsatellite status, Table S6: Multivariate overall survival analysis in the overall cohort under exclusion of CRC subtype, tumour budding and WHO grade.
