# Supplementary material for: Loss of SATB2 Occurs More Frequently Than CDX2 Loss in Colorectal Carcinoma and Identifies Particularly Aggressive Cancers in High-Risk Subgroups
Source: Cancers (Basel). 2021 Dec 7;13(24):6177. doi: 10.3390/cancers13246177 (PMC8699173; doi:10.3390/cancers13246177)
Supplement: Supplementary file 1 [file cancers-13-06177-s001.zip › cancers-1483289-supplementary.pdf]

Supplementary Materials

Loss of SATB2 Occurs More Frequently Than CDX2 Loss in Colorectal Carcinoma and Identifies Particularly Aggressive Cancers in High-Risk Subgroups

Maxime Schmitt, Miguel Silva, Björn Konukiewitz, Corinna Lang, Katja Steiger, Kathrin Halfter, Jutta Engel, Paul Jank, Nicole Pfarr, Dirk Wilhelm, Sebastian Foersch, Carsten Denkert, Markus Tschurtschenthaler, Wilko Weichert and Moritz Jesinghaus

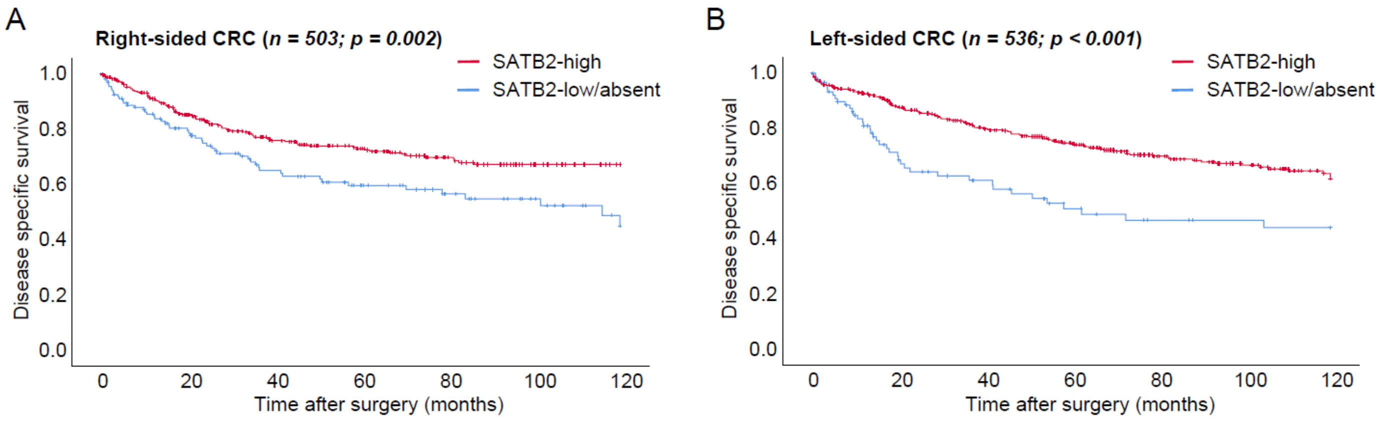

Figure S1. Prognostic relevance of SATB2 expression for disease-specific survival in right-sided (A, coecum until splenic flexure) and left-sided (descending colon until rectum) CRCs.

Table S1. Correlation between SATB2 expression groups with SATB2 staining pattern (A) and SATB2 staining intensity (B).

**Variables**	**Variables**					**Total**	***p*-Value**
A	SATB2 staining pattern						
		absent	heterogenous	diffuse			
SATB2 groups	low/absent	60	155	16		231	
	high	0	185	623		808	*p*<0.001
total		60	340	639		1039	
B	SATB2 staining intensity						
		absent	weak	medium	strong		
SATB2 groups	low/absent	60	118	48	5	231	
	high	0	27	164	617	808	*p*<0.001
total		60	145	212	622	1039	

Table S2. Distribution of SATB2 expression groups with clinicopathological and morphological parameters in the overall cohort.

Variables		Overall	SATB2 Low/Absent	SATB2 High	*p*-Value
		1039 (100%)	231 (22.2%)	808 (77.8%)	
Age					0.297
	below median	504 (48.5%)	105 (45.5%)	399 (49.4%)	
	above median	535 (51.5%)	126 (54.5%)	409 (50.6%)	
Gender					0.227
	female	440 (42.3%)	106 (45.9%)	334 (41.3%)	
	male	599 (57.7%)	125 (54.1%)	474 (58.7%)	
pT					<0.001
	1	79 (7.6%)	6 (2.6%)	73 (9%)	
	2	187 (18%)	31 (13.4%)	156 (19.3%)	
	3	578 (55.6%)	127 (55%)	451 (55.8%)	
	4	195 (18.8%)	67 (29%)	128 (15.8%)	
pN					<0.001
	0	580 (55.8%)	97 (42%)	483 (59.8%)	
	1	292 (28.1%)	69 (29.9%)	223 (27.6%)	
	2	167 (16.1%)	65 (28.1%)	102 (12.6%)	
pM					<0.001
	0	887 (85.4%)	174 (75.3%)	713 (88.2%)	
	1	152 (14.6%)	57 (24.7%)	95 (11.8%)	
UICC Stage					<0.001
	1	213 (20.5%)	27 (11.7%)	186 (23%)	
	2	350 (33.7%)	67 (29%)	283 (35%)	
	3	318 (30.6%)	78 (33.8%)	240 (29.7%)	
	4	158 (15.2%)	59 (25.5%)	99 (12.3%)	
Tumour type (WHO)					<0.001
	Adenocarcinoma NOS	650 (62.6%)	106 (45.9%)	544 (67.3%)	
	Mucinous adenocarcinoma	88 (8.5%)	36 (15.6%)	52 (6.4%)	
	Signet-ring cell carcinoma	9 (0.9%)	6 (2.6%)	3 (0.4%)	
	Medullary adenocarcinoma	32 (3.1%)	13 (5.6%)	19 (2.4%)	
	Micropapillary adenocarcinoma	129 (12.4%)	41 (17.7%)	88 (10.9%)	
	Serrated adenocarcinoma	91 (8.7%)	19 (8.2%)	72 (8.9%)	
	Adenoma-like adenocarcinoma	33 (3.2%)	5 (2.2%)	28 (3.5%)	
	MANEC/NEC	7 (0.7%)	5 (2.2%)	2 (0.2%)	
WHO grade					<0.001
	low-grade	708 (68.1%)	125 (54.1%)	583 (72.2%)	
	high-grade	331 (31.9%)	106 (45.9%)	225 (27.8%)	
Tumour budding					<0.001
	Bd1	560 (53.9%)	96 (41.6%)	464 (57.4%)	
	Bd2	270 (26%)	60 (26%)	210 (26%)	
	Bd3	209 (20.1%)	75 (32.5%)	134 (16.6%)	
Resection margin					<0.001
	R0	960 (92.4%)	199 (86.1%)	761 (94.2 %)	
	R1	49 (4.7%)	22 (9.5%)	27 (3.3%)	
	R2	30 (2.9%)	10 (4.3%)	20 (2.5%)	
Lymphatic invasion					0.001
	not present	508 (48.9%)	74 (32%)	434 (53.7%)	
	present	531 (51.1%)	157 (68%)	374 (46.3%)	
Venous invasion					<0.001
	not present	904 (87%)	179 (77.5%)	725 (89.7%)	
	present	135 (13%)	52 (22.5%)	83 (10.3%)	
Perineural invasion					0.001
	not present	956 (92%)	200 (86.6%)	756 (93.6%)	

	present	83 (8%)	31 (13.4%)	52 (6.4%)	
Microsatellite status					0.01
	Microsatellite stable	877 (84.4%)	182 (78.8%)	695 (86%)	
	Microsatellite instable	162 (15.6%)	49 (21.2%)	113 (14%)	
Tumour localization					<0.001
	Right colon (Coec/Asc/Trans)	503 (48.4%)	138 (59.7%)	365 (45.2%)	
	Left colon (Desc/Sigm/Rect)	536 (51.6%)	93 (40.3%)	443 (54.8%)	

Table S3. Impact of SATB2 expression on overall, disease-specific and disease-free survival in tumour budding (Bd1, Bd2, Bd3), WHO grade (low, high), microsatellite (MSS, MSI-H) and CDX2 expression subcohorts (low/absent, high).

Variables	SATB2 Groups	Overall *n* (%)	Mean Overall Survival (SE) [Months]	*p*-Value	Mean Disease Specific Survival (SE) [Months]	*p*-Value	Mean Disease Free Survival (SE) [Months]	*p*-Value
WHO grade: low-grade subcohort				0.110		0.027		0.001
	SATB2 low/absent	125 (17.6%)	81.1 (4.7)		88.1 (4.7)		77.1 (5.1)	
	SATB2 high	583 (82.4%)	87.1 (2.0)		96.7 (1.9)		92.1 (2.0)	
WHO grade: high-grade subcohort				0.013		0.002		0.016
	SATB2 low/absent	106 (32%)	54.8 (5.0)		59.5 (5.3)		57.4 (5.6)	
	SATB2 high	225 (68%)	70.3 (3.4)		78.8 (3.5)		72 (3.6)	
Tumour budding: Bd1 subcohort				0.034		0,05		0.026
	SATB2 low/absent	96 (17.1%)	90.3 (4.8)		104.4 (3.9)		99.7 (4.6)	
	SATB2 high	464 (82.9%)	99.3 (1.9)		110.2 (1.4)		108.3 (1.6)	
Tumour budding: Bd2 subcohort				0.147		0.564		0.258
	SATB2 low/absent	60 (22.2%)	78.3 (6.7)		80.7 (6.7)		74.7 (7.2)	
	SATB2 high	210 (77.8%)	68.4 (3.5)		76.7 (3.6)		64.6 (3.8)	
Tumour budding: Bd3 subcohort				0.005		0.002		<0.001
	SATB2 low/absent	75 (35.9%)	29.4 (4.4)		29 (4.3)		19.4 (3.6)	
	SATB2 high	134 (64.1%)	46.7 (4.0)		51.1 (4.4)		44.3 (4.4)	
Microsatellite status: MSS subcohort				<0.001		<0.001		<0.001
	SATB2 low/absent	182 (20.7%)	65.4 (4.0)		70 (4.1)		62.8 (4.3)	
	SATB2 high	695 (79.3%)	80.7 (1.9)		89.4 (1.8)		84.1 (2.0)	
Microsatellite status: MSI-H subcohort				0.072		0.023		0.163
	SATB2 low/absent	49 (30.2%)	78.8 (7.3)		90.6 (7.1)		89 (7.9)	
	SATB2 high	113 (69.8%)	92.3 (4.4)		105.8 (3.6)		101 (4.19)	
CDX2 low-absent subcohort				0.026		0.021		0.0069
	SATB2 low/absent	64 (60.4%)	58.4 (7.2)		64.5 (7.6)		60.1 (7.6)	
	SATB2 high	42 (39.6%)	82.1 (8.2)		91.2 (7.8)		84.6 (8.5)	
CDX2 high subcohort				0.013		<0.001		<0.001
	SATB2 low/absent	167 (17.9%)	71.8 (4.0)		77.3 (4.1)		70.7 (4.5)	
	SATB2 high	766 (82.1%)	82.2 (1.8)		91.6 (1.7)		86.5 (1.9)	

Table S4. Impact of SATB2 expression on overall, disease-specific and disease-free survival in the different UICC stage groups.

Variables	SATB2 Groups	Overall *n* (%)	Mean Overall Survival (SE) [Months]	*p*-Value	Mean Disease Specific Survival (SE) [Months]	*p*-Value	Mean Disease Free Survival (SE) [Months]	*p*-Value
UICC stage I				0.397		0.593		0.608
	SATB2 low/absent	27 (12.7%)	93.1 (8.2)		111.1 (5.3)		114.6 (4.6)	
	SATB2 high	186 (87.3%)	97.1 (3.1)		111 (2.3)		107 (2.6)	
UICC stage II				0.977		0.664		0.342
	SATB2 low/absent	67 (19.1%)	86.2 (6.4)		95.5 (6.2)		89.5 (6.7)	
	SATB2 high	283 (80.9%)	86 (2.6)		97.3 (2.7)		96.9 (2.8)	
UICC stage III				0.025		0.012		0.004
	SATB2 low/absent	78 (24.5%)	70.3 (6.1)		74.5 (6.2)		62.1 (6.5)	
	SATB2 high	240 (75.5%)	84.4 (3.1)		91 (3.0)		81.2 (3.4)	
UICC stage IV				0.593		0.397		0.564
	SATB2 low/absent	59 (37.3%)	37.4 (5.6)		38.3 (5.7)		33.4 (6.2)	
	SATB2 high	99 (62.7%)	40.3 (4.2)		43.9 (4.6)		33.2 (3.5)	

Table S5. Multivariate overall survival analysis in the overall cohort under inclusion of SATB2 expression, age, gender, CRC subtype, tumour budding, WHO grade and microsatellite status.

Variables		HR (DSS)	Lower CI (95%)	Upper CI (95%)	*p*-Value
SATB2 subgroups					0.1
	SATB2 high	1.00			
	SATB2 Low/absent	1.25	0.95	1.63	
WHO Subtype					0.05
	Adenocarcinoma NOS	1.00			
	Mucinous adenocarcinoma	0.99	0.63	1.56	
	Signet-ring cell carcinoma	0.88	0.31	2.53	
	Medullary carcinoma	0.14	0.02	1.01	
	Micropapillary adenocarcinoma	0.88	0.65	1.19	
	Serrated adenocarcinoma	1.11	0.72	1.71	
	Adenoma-like adenocarcinoma	0.69	0.17	2.88	
	MANEC/NEC	2.16	0.84	5.53	
Tumour budding					<0.001
	Bd1	1.00			
	Bd2	3.14	2.23	4.42	
	Bd3	6.73	4.65	9.74	
WHO-grade					0.016
	Low grade	1.00			
	High grade	1.36	1.06	1.74	
UICC Stage					<0.001
	I	1.00			
	II	1.65	0.99	2.76	
	III	1.73	1.04	2.89	
	IV	3.89	2.26	6.70	
Gender					0.293
	female	1.00			
	male	1.14	0.89	1.45	
Resection status					0.001
	R0	1.00			
	R1/2	1.43	1.17	1.77	

Tumour localization					0.426
	Right colon	1.00			
	Left colon	1.10	0.86	1.41	
Age group					0.007
	Below median	1.00			
	Median and above	1.39	1.10	1.78	
Microsatellite status					0.9
	Microsatellite instable	1.00			
	Microsatellite stable	1.00	0.63	1.59	

Table S6. Multivariate overall survival analysis in the overall cohort under exclusion of CRC subtype, tumour budding and WHO grade.

Variables		HR (DSS)	Lower CI (95%)	Upper CI (95%)	*p*-Value
SATB2 subgroups					0.029
	SATB2 high	1.00			
	SATB2 Low/absent	1.32	1.03	1.71	
UICC Stage					<0.001
	I	1.00			
	II	2.58	1.57	4.25	
	III	3.64	2.24	5.91	
	IV	10.36	6.25	17.17	
Gender					0.85
	female	1.00			
	male	1.14	0.93	1.50	
Resection status					<0.001
	R0	1.00			
	R1/2	1.61	1.31	1.98	
Tumour localization					0.426
	Right colon	1.00			
	Left colon	1.10	0.86	1.41	
Age group					<0.001
	Below median	1.00			
	Median and above	1.54	1.22	1.95	
Microsatellite status					0.029
	Microsatellite stable	1.00			
	Microsatellite instable	0.6	0.40	0.95	
